# Utilisation of Waste Sludge from Drinking Water Treatment as a Filler Material in Hot Mix Asphalt

**DOI:** 10.3390/ma17071528

**Published:** 2024-03-27

**Authors:** Tuna Eyüp Kahveci, Halit Özen

**Affiliations:** 1 Department of Civil Engineering, Yildiz Technical University, İstanbul 34220, Turkey; 2 Yıldız Teknik Üniversitesi İnşaat Müh. Bölümü, YTÜ-Davutpaşa Kampüsü, İstanbul 34220, Turkey; ozen@yildiz.edu.tr

**Keywords:** sludge, HMA, waste, filler

## Abstract

This research investigated the suitability of using sludge from the treatment of drinking water in hot mix asphalt (HMA) as a filler material. The storage and environmental impact of sludge is an enormous problem, especially for countries with large populations. Two different types of sludges, ferric chloride (FC) and aluminium sulphate (AS), were used as a filler material in HMA. The Hamburg Wheel Tracking (HWT) test, which correlates with rutting, and the Indirect Tensile Strength (ITS) test, which indicates the moisture sensitivity of HMA, were carried out at the optimum bitumen content of the mixes to investigate the usability of sludge in HMA. The test results indicate the usability of FC and AS in HMA compared to the reference mixes. However, the AS type of sludge has better rutting resistance than the FC type. Although the results support the usability of both sludges in HMA, it should be noted that the increased cost of the mix containing sludges due to the combustion process and the increased bitumen content during application should be considered.

## 1. Introduction

The Turkish State Water Affairs Institution in the National Water Plan 2019–2023, 2018 [1] indicates that the average annual rainfall volume in the Republic of Turkey is 450 billion m^3^, and the total annual consumable surface and groundwater potential is 112 billion m^3^. As a result of the projects developed by public institutions and organizations responsible for the development of the country’s land and water resources, annual water consumption for various purposes has reached 54 billion m^3^ (48.2%) of this water, 40.0 billion m^3^ (74%) is used for irrigation, 7 billion m^3^ (13%) is used for drinking use, and 7 billion m^3^ (13%) is used to meet industrial water needs. For 2017, 39 billion m^3^ (72.2%) of the consumed water was provided by surface water, and 15 billion m^3^ (27.8%) by groundwater.

When calculated using the 2017 address-based population data published by the Turkish Statistical Institute (TurkStat) Municipal Water indicators, Water and Wastewater Statistics 2020 [2], it is seen that the annual amount of usable water per capita of Turkey is around 1400 m^3^.

According to the Turkish Statistical Institute data in 2020 [2] mentioned above, 3 billion 900 million m^3^ of the drinking water used annually in the Republic of Turkey has been treated. The relation between used and treated drinking water is shown in Table 1.

The amounts of water treated and delivered to users and produced sludge listed in Table 2.

These quantities correspond to approximately 27.5% of the drinking water treated throughout Turkey for 2020. Table 3 shows the amounts of treated water, produced sludge, and average solid matter in sludge by the facilities of the İstanbul Water and Sewerage Administration (ISKI). The average solid matter in sludge was only given in the 2018 and 2019 activity reports [3,4]. For the following years, these amounts are not indicated in the reports.

It can be seen from the calculations that the approximate annual produced solid sludge is around 126,629,630 tons in Turkey; by treating the total annual drinking water amount, it has the potential to reach 233,000 tons per year. The studies about drinking water treatment sludge (DWTS) show that DWTS can be used in many sectors, like geotechnical, water, and environmental engineering areas, agriculture, construction materials, coagulants in wastewater treatment, and as a remover or purificator of some hazardous materials.

The top layers in a flexible pavement are constructed as HMA, which is a mixture of graded mineral aggregates and bitumen. Bitumen is a brown to black solid (or semi-solid at ambient temperature) substance, which is a mixture of heavy hydrocarbons and their derivatives [8]. Bitumen is commonly used as a binder for bituminous mixes in the paving sector. There are other uses for asphalt, such as under railway tracks [9] or in insulation materials. Aggregates in HMA are graded as coarse, fine, and filler. Fillers occupy between 5 and 12% of asphalt mixture and are very fine materials that mostly pass sieve sizes of 0.063 mm or 0.075 mm depending on the standard used. In contrast to its small amount in a bituminous mixture, its contribution to the physical and chemical properties of the material is significant [10]. Various types of waste such as high-density polyethylene, marble quarry waste, building demolition waste, ground tyre rubber, cooking oil, palm oil fuel ash, coconut, sisal, cellulose and polyester fibres, starch, plastic bottles, waste glass, waste bricks, waste ceramics, waste fly ash, and cigarette butts are recycled in asphaltic concrete or in bitumen [11]. There are two main approaches used to waste in HMA. One of them is the incorporation of waste materials into bitumen at high temperatures by mechanical mixing [12,13]. The other one is adding waste directly to the mixture of bitumen and aggregates, either as a partial aggregate replacement or a mixture modifier [14]. In this study, waste was added into HMA as a partial aggregate replacement.

According to the studies conducted so far, some of the areas of the use of DWTS can be summarized as follows. DWTS presents a valuable and environmentally sound solution across various fields. Balkaya [15] indicates that in the area of soil enhancement, it emerges as a promising additive that can effectively improve soil quality. This extends to geotechnical applications, as indicated in the article published by Boscov et al. [16] in which it stands out as a sustainable alternative to preserve natural soils. Sabo, A et al. [17] indicate that the absence of excessive toxic metals in DWTSs, supply of abundant plant nutrients, and lack of harmful toxic metals, emphasizes its viability as a valuable resource for enriching soil nutrients during the reclamation of degraded lands. Verlicci and Masotti [18] show that DWTS establishes that its usefulness in aquatic ecosystems, such as rejuvenating eutrophic lakes, is noteworthy, offering a potent remedy to restore these imbalanced environments. Moreover, it presents with functionality in activities like pit filling, reclamation, and capping waste landfills, thereby contributing to effective waste management. Maria et al. [19] present, for the first time, the results of the successful application of the waste press sludges, WSLP (plant for lacquer and paint) and WSEP (powdery enamel plant), from a wastewater treatment plant. These wastes were generated during heating device production in the construction industry. Zhao et al. [20] demonstrate that DWTS finds its place as a substrate in developing constructed wetland treatment systems. Kevin et al. [21] and Liu et al. [22] revealed in their studies that, according to the analysis of the samples, SiO_2_, Al_2_O_3_, and Fe_2_O_3_ represent 90% of the composition of sludge ash which, according to ASTM C618-17a [23], classifies sludge ash as pozzolan material class F, showing the potential of the DTWS as a supplementary cement material. Noruzman et al. [24] observed in their research that sludge in brick performed better when mixed using 5% as a partial replacement of sand. However, the addition of a higher percentage of sludge in brick, resulted in lower strength. It can be concluded that waste treatment sludge, because of the process of water treatment, can be utilized as a partial replacement for sand in brick production.

It also can be used to construct barrier layers, and the formation of “bio-soils” reduces the consumption of natural materials and the demand for landfill volumes and offers numerous technological advantages, as reported by Caniani et al. [25] in their article. Results from Liu and Zhuge [26] imply that concrete paving blocks, for instance, benefit from waste treatment sludge incorporation, as it enriches their structural integrity and performance. Additionally, Kizinievic, O and Kizinievic, V [27] prove that the synthesis of ceramic products finds a novel dimension with the inclusion of DWTS, which amplifies their quality and utility. The results of Sarabia-Guarin et al. [28] indicate that DWTS has a role in manufacturing refractory bricks, where it serves as a judicious partial substitute for clay, bolstering the efficiency of the production process. Liu and Zhuge [26], in their study, prove that high alumina content within DWTS may endow sludge-derived concrete products with superior properties, such as a higher resistance to fire and alkali-silica reactions, harnessing the value of alumina in sludge. This allows the water industry to valorise its waste and gain more commercial benefits. Zandy et al. [29] and Sofy et al. [30] indicated in their two different studies that DWTS and activated drinking water treatment sludge seemed to be suitable for producing blended cement at 5–10% increased levels and for use in concrete. The study of Rodrigues et al. [31] confirms that DWTS reduces energy expenditure due to drying and grinding in ceramic production.

Zhao et al. [20] demonstrate that, as alum sludge, DWTS plays a crucial role in purifying unpleasant gases, mitigating their impact on the environment. Its efficiency as an affordable adsorbent is engaged in immobilizing diverse pollutants and contributing to pollution control efforts. Nuria et al. [32] indicate that in the domain of wastewater treatment, DWTS serves as an active substrate within vertical subsurface flow-constructed wetlands (VFCW), constituting a powerful tool for advanced wastewater treatment. El Eneina et al. [33] expressed, based on the results in their article, that its ability to adsorb heavy metals, and thus, to act as an efficient agent for environmental remediation, is especially noteworthy. Zhao [34] states in his article that the development of alum sludge-based constructed wetland systems as an innovative approach not only enhances the removal of organic matter and nutrients from high-strength wastewater but also showcases DWTS’s adaptability to address complex environmental challenges. Kucukcongar et al. [35] indicate that DWTS plays a pivotal role as a coagulant in wastewater treatment, aggregating impurities for efficient removal and contributing to improved water quality. Given the substantial magnitude of this sludge production, it becomes imperative to conduct thorough research to identify and explore viable applications for the by-products of drinking water treatment. This study aims to investigate the use of DWTS as a component of HMA. This study is expected to lead the domains of both waste management and construction materials toward a more efficient and eco-friendlier environment.

## 2. Materials

In this study, “Wearing Course Type 1”, defined as HMA in KTS 2013 [36], Cumhuriyet sludge, referred to in the text as “FC”, and Ikitelli sludge, referred to in the text as “AS”, were used to test the usability of sludges. The detailed bitumen and aggregate information and specification limits for them are given in the following sections. For the materials, all tests were done in the certified laboratory of İSFALT A.Ş., which is the establishment of İstanbul Greater Municipality.

### 2.1. Bitumen

The AC 50/70 bitumen that was used in the study was obtained from the TÜPRAŞ İZMİT refinery in Kocaeli, Turkey. Properties of bitumen and the specification limits according to Technical Specification for Türkiye Highway Directorate (KTS 2013) [36] are given in Table 4.

### 2.2. Aggregate

**Figure 1:**
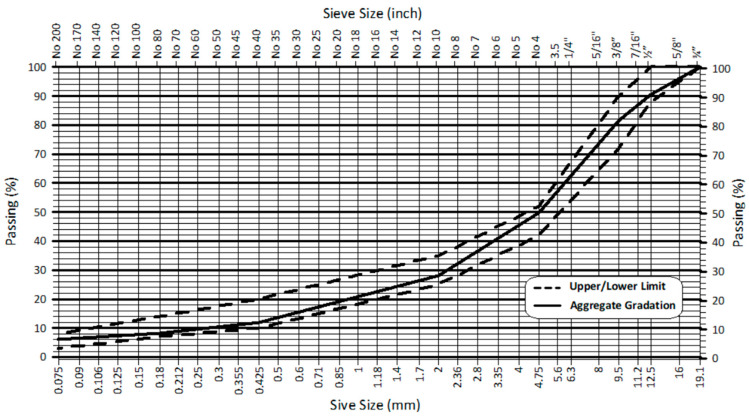
Aggregate gradation and limits.

Limestone, used as an aggregate in the study, was obtained from the Koc Stone Querry in three different aggregate sizes: 19–37.5 mm, 12–19 mm, and 5–12 mm. Aggregate mixture gradations were made using the proportions of 9, 48, and 43% for 19–37.5 mm, 12–19 mm, and 5–12 mm aggregates, respectively, and specification limits according to KTS 2013 [36] for Wearing Course Type 1 are listed in Table 5 and shown in Figure 1. The gradation of FC is presented in the same table.

Physical properties tests were conducted on the aggregates, and the result of the tests are presented in Table 6.

### 2.3. Mixture

In this study, conventional Wearing Course Type 1, as defined in KTS 2013 [36], was used as a reference HMA to compare the performance measure of the other slugged added HMA. The reference HMA optimum bitumen content was based on the Marshal mix design method (ASTM 1559 [47]), using aggregate and bitumen that had the properties of the material given in the previous section. In the design of the reference HMA, samples with a different bitumen content were compacted with a Marshal Compacter by implementing 75 below on each side of cylindrical samples at a temperature between 135 and 140 °C. Brief results for the Marshall method are shown in Table 7. As seen in Table 7, the optimum bitumen content for the reference HMA was determined as Wa = 4.60%, and the Marshall stability was 1300 kg, more than the specification limit of KTS 2013 [36].

### 2.4. Drinking Water Treatment Sludge (DWTS)

In this research, sludge treated with two different chemicals, FC (%40) (Ferric Sludge) and AS (%50) (Alum Sludge) and supplied by İSKİ Cumhuriyet (Beykoz, Turkey) and İSKİ İkitelli (İstanbul, Turkey) drinking water treatment plants was used as waste. Both types of DWTS properties are given in Table 8.

**Figure 2:**
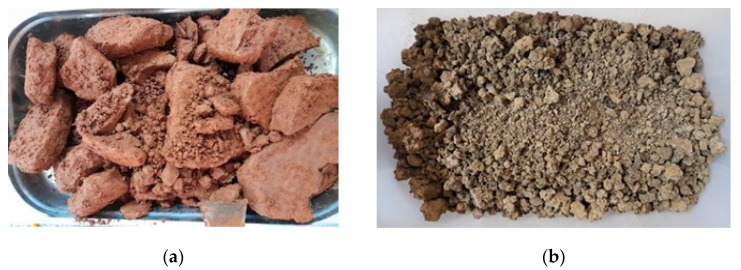
Picture of DWTS samples from İSKİ Cumhuriyet and Ikitelli Water Treatment Plants. (**a**) DWTS sample (FC) obtained from İSKİ Cumhuriyet Plant. (**b**) DWTS sample (AS) obtained from İSKİ Ikitelli Plant.

DWTS, obtained from the İSKİ Cumhuriyet Drinking Water Treatment Plant, was processed with %40 concentrated Ferric (III) chloride solution. As a result, the colour of the material is almost red (Figure 2a). The DWTS obtained from the İSKİ İkitelli Drinking Water Treatment Plant was processed with %50 concentrated Aluminium Sulphate solution and the colour of that sample is almost green (Figure 2b).

**Figure 3:**
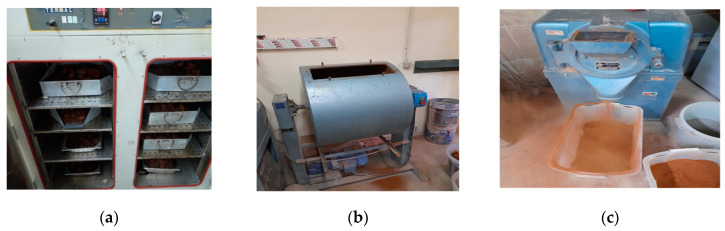
Preparation process of DWTS sample for HMA. (**a**) Drying DWTS in oven at 150 °C. (**b**) Cutting into pieces using Los Angles Abrasion Test Equipment, Utest, Ankara, Türkiye. (**c**) Grinding and crumbling of dried DWTS.

DWTS supplied from the facilities was prepared as described. First, the DWTS was dried at 150 °C in the oven at the Yıldız Technical University (YTU) Transportation laboratory (Figure 3a). The weights of the tared sample containers were determined for both types of sludge separately before the drying process started. These sample containers were weighed regularly during drying, and the amount of liquid loss, depending on the time, was determined. The drying process was terminated when it was concluded that the liquid loss was over. Thus, the solid waste amounts and drying times of both types of DWTS were also determined. After finishing the drying process, DWTS was cut into small pieces using balls in the Los Angeles experimental sphere separately in the Building Materials laboratory (Figure 3b), and these shrunken pieces were passed through the grinder and turned into filler material (Figure 3c). Humidity was calculated, after the drying process in the oven, at % 58.3 and %76.3, respectively, for FC and AS DWTS.

**Figure 4:**
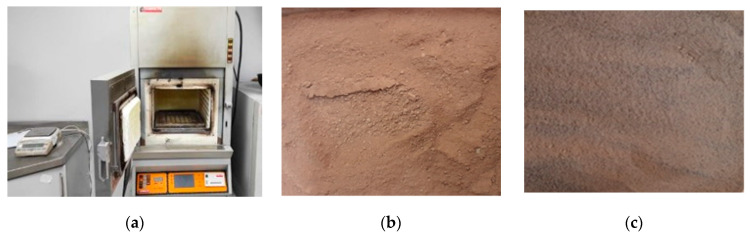
Burning process and final sample of DWTS to use in HMA. (**a**) Burning DWTS in oven at 500 °C. (**b**) FC DWTS filler. (**c**) AS DWTS filler.

The sludge, which was ground into filler material, was subjected to a new process in the facilities of İSFALT A.Ş., ELE, Milton Keynes, UK to remove the organic substances in it, burned in an oven at 500 °C, as seen in Figure 4a, and made available to use as an additive for HMA. Figure 4b,c present FC and AS DWTS ready to use in HMA. The amount of organic substances was calculated after the burning process in the furnace oven, and was found to be %10.4 and %20.1, respectively, for FC and AS DWTS.

## 3. Experimental Methods

In this research, DWTS was used as a filler by adding it to aggregate for HMA; as a result, the gradation of the DWTS added to the aggregate mixture was changed. The amount of the added waste was reduced from the aggregate 5–12 mm, as seen in Table 5. Based on this, the filler in the aggregate mixture with waste was calculated as 6.9%, 7.2%, and 7.4% for 1%, 3%, and 5% DWTS as a replacement of 5–12 mm aggregate. Increasing the amount of DWTS in aggregates causes an increase in filler, and the amount of filler becomesclose to the specification limit, which is 8% for filler in aggregates. Initial laboratory tests such as the Marshall stability (ASTM 1559 [47]) and indirect tension tests (AASHTO T283 [48]) were performed to calculate the tensile strength ratio for 4.6% bitumen content to find the proper amount of DWTS for HMA. Initial laboratory tests were conducted only with FC DWTS mixture to determine the appropriate amount of waste in HMA. The results of the tests are presented in Table 9.

In this study, the amount of DWTS is determined as 3% because of the following:

- Fillers have more surface area per unit volume, therefore increasing the amount of filler in the mixture will require more bitumen in the mixture, thus increasing the cost of HMA with added DWTS.
- HMA tends to have rutting potential because of the excessive void.
- Increased DWTS causes decreased TSR.
- The Marshall stability for a 3% DWTS mixture is greater than for the other DWTS amounts.

The initial test indicated that bitumen content is important for the HMA with DWTS. Therefore, the Marshall mix design method was applied for both FC and AS mixtures to determine the optimum bitumen content of HMA with DWTS. The HMA with DWTS Marshall mixture design method parameters are presented in Table 10.

The indirect tensile test is one of the most popular methods to evaluate the effect of moisture that causes stripping on compacted asphalt mixtures. The test was carried out according to AASHTO T283 [49] to assess the ITS of compacted asphalt mixtures in dry and wet conditions. The equation below defines the ratio of the ITS of conditioned and unconditioned samples as the tensile strength ratio (TSR). In the equation, ITS is the indirect tensile strength of HMA in (kN).

(1) $$TSR=\frac{{ITS}_{conditioned}}{{ITS}_{unconditioned}}\times 100$$

The ITS test results for all mixtures are given in Table 11.

The HMA sample’s rutting resistance was evaluated using the HWT test. The HWT test was carried out by following the AASHTO T 324-11 standard [49]. The sample is subjected to the steel rolling wheel with a 52 passes/min rate. A recording by a Linear Variable Differential Transformer (LVDT) was obtained for both the specimens’ left and right sides after each pass’s completion. The test concludes automatically when the specimen undergoes 20,000 passes or reaches a maximum vertical deformation of 20 mm. The air voids of HMA specimens were controlled at 7 ± 1%

**Figure 5:**
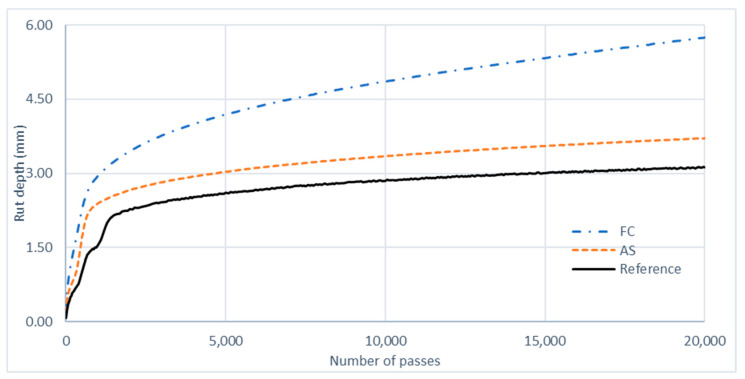
Rutting curve for HMA mixtures.

After HWT testing, a rutting data curve, shown in Figure 5, was generated for each testing sample. Table 12 presents the results of HWT testing and the calculated values of the wheel-tracking slope.

## 4. Evaluation

In this research, Marshal mix designs, indirect tensile tests, and HWT tests at the optimum bitumen content were conducted for the reference mixture, FC mixture, and AS mixture. The performance of the mixtures was evaluated for stability by the Marshall method, the effect of moisture by ITS, and rutting resistance by the HWT test.

**Figure 6:**
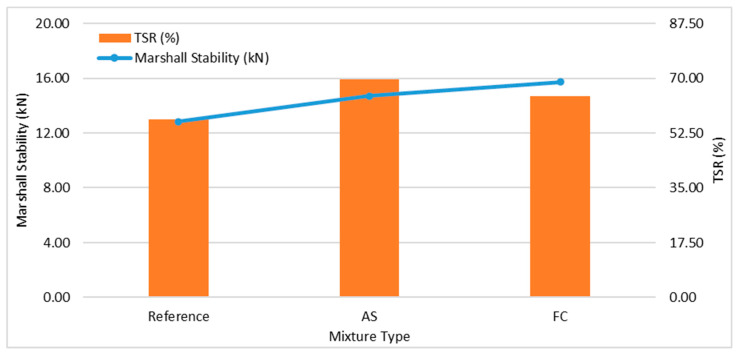
Marshall stability (kN) and TSR (%) values for the mixtures.

Marshall and ITS test results are summarized in Table 13 and depicted in Figure 6 as a graph. As shown in Table 13, mixture FC had the most robust Marshall stability, with a Marshall stability of 15.73 kN, distinctly outperforming mixture AS, which had a Marshall stability of 14.71 kN, and the reference mixture, which had a Marshall stability of 12.82 kN.

An examination of Figure 6 shows that both mixture AS and mixture FC exhibit superior performance in terms of TSR values compared to the reference mixture. When mixture FC and mixture AS are compared, it is observed that mixture AS has better TSR values than mixture FC.

The performance of tested asphalt mixtures from the HWT test is typically described using a deformation evolution curve divided into three stages. The primary or post-compaction stage is the initial stage and is usually within the first 1000 wheel passes. This stage is followed by the secondary stage, which captures the creeping region of the asphalt mixture. Finally, the tertiary stage is related to the moisture susceptibility performance of the asphalt mixture. The final rut depth of the asphalt mixture is typically associated with the resistance of the asphalt mixture to rutting when tested using the HWT. As shown in Figure 5, all of the HMA mixtures likely experienced the post-compaction and creep phases, and the striping phase did not occur before 20,000 passes during the test.

The rut depth of mixture FC is likely more than that of the reference mixture and mixture AS. The post-compaction phase occurs before the reference mixture for mixtures FC and AS, even if the rut depth is low (Figure 5).

**Figure 7:**
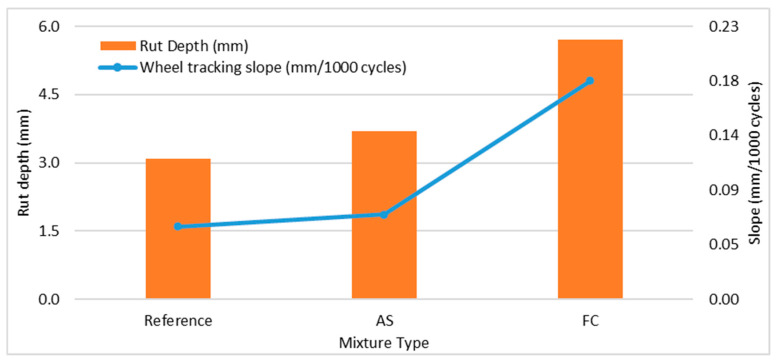
HWT test values of rut depth and creep slope for mixtures.

Figure 7 presents rut depth at the end of the HWT test, which is at 20,000 passes, and wheel tracking slope, which is the creep phase slope of the mixtures. As seen in Figure 7, the reference mixture and mixture AS rut depth at 20,000 passes are close, and less than mixture FC. The creep phase slopes of the mixtures display a similar trend for rut depth at 20,000 passes.

## 5. Conclusions

In this research, the Hamburg Wheel Tracking Test (HWT), which is correlated with rutting, and the indirect tensile strength (ITS) test, which indicates the moisture sensitivity of HMA, were conducted at the optimum bitumen content of the mixture to investigate the usability of sludge in HMA. Two types of sludges, based on applying ferric chloride (FC) and aluminium sulphate (AS), were used. The permanent deformation, stripping, and moisture sensitivity of the reference mixture, FC mixture, and AS mixture at the optimum bitumen content were investigated.

Mixture FC had the most robust Marshall stability, distinctly outperforming mixture AS and the reference mixture. The Marshall stability of the mixtures with both types of sludges was more than the reference mixture. Both mixture AS and mixture FC exhibited superior performance in terms of TSR values when compared to the reference mixture. When mixture FC and mixture AS are compared, it is observed that mixture AS had better TSR values than mixture FC.

All of the HMA mixtures likely experienced the post-compaction phase and creep phase, and the striping phase did not occur before 20,000 passes during the test. The rut depth of mixture FC is likely more than that of the reference mixture and mixture AS. The slope of the creep stage corresponds to the mixture rutting resistance, while the slope of the stripping stage is related to the stripping resistance. The slopes are higher, indicating less rutting resistance of the mixtures. Therefore, it can be said that mixture AS had a better rutting resistance than mixture FC.

Finally, the testing results indicate FC and AS usability in HMA compared to the reference mixtures. However, the AS type of sludge has a rutting resistance superior to that of the FC type of sludge. Although the results are favourable for the usability of both sludges in HMA, it should be noted that the increase in the cost of the mixture with sludges, due to the burning process and the increase in bitumen content during application, should be considered. As a further study, it would be interesting to look at the effects of the waste when it is added directly to the bitumen.

## Figures and Tables

**Table 1:** Water and Wastewater Statistics 2020, Turkish Statistical Institute TURKSTAT [2].

Indicator	2018	2020
Total Number of Municipalities	1399	1389
Total amount of water drawn by drinking and potable water network (million m^3^)	6193	6492
Amount of water treated in drinking and potable water treatment plants (million m^3^)	3574	3900
Average amount of water withdrawn per person (liter/person-day)	224	228

**Table 2:** Drinking Water and Sludge Produced by İSKİ [3,4,5,6,7].

Year	Amount of Water Treated (Million m^3^)	Amount of Sludge Produced (kg)	The Amount of Water Treated to Obtain 1 kg of Sludge (kg)
2018	1,040,965	89,226,113	11.67
2019	1,061,770	102,507,300	10.36
2020	1,074,134	132,538,070	8.10
2021	1,073,990	128,507,880	8.36
2022	1,103,672	121,588,820	9.08

**Table 3:** Sludge Produced as a Waste Product in ISKI Drinking Water Treatment Plants [3,4].

Name of Plant	Sludge (kg/Year)			Average Solid Matter Ratio (%)		
	2018	2019	2020	2018	2019	2020
Buyukcekmece	6,012,573	5,300,000	6,500,330	17.91	17.58	Unspecified
Kagithane	19,154,420	16,841,030	11,864,190	18.62	19.31	Unspecified
Ikitelli	20,349,190	24,043,270	23,335,120	19.01	18.20	Unspecified
Tasoluk	620,700	671,000	754,280	18.68	18.23	Unspecified
Omerli	4,710,610	18,173,400	25,439,050	30.00	30.00	Unspecified
Cumhuriyet	38,378,620	37,478,600	64,645,100	36.01	35.13	Unspecified
Total	89,226,113	102,507,300	132,538,070	27.06	26.30	Unspecified

**Table 4:** Properties of Bitumen.

Test	Test Method	Unit	Test Result	Specification Limits
Penetration	ASTM D-5 [37]	0.1 mm	53	50–70
Specific Gravity d25/25	ASTM D-70 [38]	gr/cm^3^	1.028	
Softening point	ASTM D-36 [39]	°C	48.6	46–54
Cleveland Flash Point	ASTM D-92 [40]	°C	292	≥230
Solubility	ASTM D-2042 [41]	%	100	≥99.0

**Table 5:** Aggregate gradation and limits in research.

Sieve Size		Specification Limits (Passing %)	Aggregate Gradation (Passing %)				Gradation of FC (%)
(Inch)	(mm)		19–37.5 mm	12–19 mm	5–12 mm	Mixture	
¾″	19	100	100	100	100	100	
½″	12.5	88–100	18	99	100	92.1	
3/8″	9.5	72–90	1	84	98	82.4	
No 4	4.75	42–52	1	16	98	49.7	100.0
No 10	2.00	25–35	1	5	61	28.8	98.1
No 40	0.425	10–20	1	3	24	11.9	54.2
No 80	0.180	7–14	1	3	17	8.7	39.9
No 200	0.075	3–8	1	2	13	6.8	26.2

**Table 6:** Physical properties of aggregates used in HMA.

Test Name	Test Method	Unit	Test Result
L.A. abrasion	ASTM C-131 [42]	%	16
Flatness index (for 5–12 mm aggregate) (Sample weight 2411.8 g)	BS 182 (part 105) [43]	%	16
Flatness index (for 12–19 mm aggregate) (Sample weight 3848.5 g)	BS 182 (part 105) [43]	%	11
Specific gravity—dry	TS EN 1097-6 [44]	g/cm^3^	2.72
Water absorption in coarse aggregates	TS EN 1097-6 [44]	%	0.44
Stripping resistance	ASTM D-1664 [45]	%	70
Clay lumps and friable aggregates	ASTM C-142 [46]	%	0
Saturated dry aggregate density (4–31.5 mm aggregate)	TS EN 1097-6 [44]	g/cm^3^	2.73

**Table 7:** Reference HMA design results.

Properties	Result	Specification Limits
Bitumen content, (%) by weight	4.6 ± 0.2	4.0–7.0
Practical specific gravity, g/cm^3^	2.438	
Marshall stability, kg	1300	Min 900
Flow, mm	3.1	2–4
Void, %	4.0	3–5
Void filled with asphalt (bitumen), %	70.0	65–75
Void in mineral aggregates (VMA), %	14.0	14–16

**Table 8:** Typical features of drinking water treatment sludge.

Parameters	AS Sludge	FC Sludge
Aluminium (% dry weight)	29.7 ± 13.3	10.0 ± 4.8
Ferric (% dry weight)	10.2 ± 12	26.0 ± 15.5
Calcium (% dry weight)	2.9 ± 1.7	8.32 ± 9.5
Magnesium (% dry weight)	0.89 ± 0.8	1.6
SiO_2_ (% dry weight)	33.4 ± 26.2	N/A
Zinc (mg/kg)	33.9 ± 28	18.7 ± 16
Lead (mg/kg)	44.1 ± 38.2	19.3 ± 25.3
Cadmium (mg/kg)	0.5	0.48 ± 0.26
Nickel (mg/kg)	44.3 ± 38.4	42.9 ± 39.2
Copper (mg/kg)	33.72 ± 32.5	18.7 ± 25.8
Chromium (mg/kg)	25.0 ± 20.1	25.7 ± 21.6
Cobalt (mg/kg)	1.06	1.61 ± 1.1
p (% dry weight)	0.35	0.36
Total solids (mg/L)	(2500–52,345)	(2132–5074)
pH	7.0 ± 1.4	8.0 ± 1.6
BOI5 (mg/L)	45 (2–104)	N/A

**Table 9:** Initial laboratory test results for determining the amount of DWTS in HMA.

Amount of DWTS (FC)	Mix Density (g/cm^3^)	Void (%)	Void Filled with Asphalt (%)	Flow (mm)	Stability (kg)	Tensile Strength Ratio (TSR) (%)	Amount of Filler in Mixture (%)
Reference	2.428	4.00	69.00	3.10	1300	56.70	6.8
[1]	2.400	5.11	64.09	3.17	1384	37.10	6.9
[3]	2.380	6.33	60.04	2.66	1487	34.60	7.2
[5]	2.350	7.11	55.89	2.30	1772	32.17	7.4

**Table 10:** Marshall mix design method results for HMA with FC and AS additions.

Parameters	Mixture Type		
	Reference	FC	AS
Optimum bitumen content (%)	4.6	5.7	6.3
Mix density (g/cm^3^)	2.418	2.401	2.381
Void (%)	4.69	3.89	3.95
Void in mineral aggregate (%)	14.35	15.82	17.02
Void filled with asphalt (%)	67.32	75.39	76.76
Marshall stability (kN)	12.82	15.73	14.71
Flow (mm)	3.54	3.64	3.46

**Table 11:** Result of ITS laboratory tests according to AASHTO T283.

HMA Type	Properties	Unconditioned Samples			Conditioned Samples		
		1	2	3	1	2	3
Reference	Void (%)	7.671	7.606	7.579	7.652	7.611	7.289
	Water absorption (%)				70.225	70.017	70.181
	ITS (kN)	166.09	162.74	147.64	98.59	80.76	90.97
	Average ITS (kN)	158.82			90.11		
	TSR (%)	56.73					
AS	Void (%)	7.138	7.007	6.932	7.040	7.076	6.751
	Water absorption (%)				70.218	70.142	70.464
	ITS (kN)	174.47	173.50	172.77	120.67	119.97	121.83
	Average ITS (kN)	173.58			120.83		
	TSR (%)	69.61					
FC	Void (%)	6.658	6.704	6.808	7.005	6.777	6.854
	Water absorption (%)				71.138	71.454	71.009
	ITS (kN)	169.23	164.97	178.11	113.62	109.09	106.65
	Average ITS (kN)	170.77			109.79		
	TSR (%)	64.29					

**Table 12:** HWT test results for HMA mixtures.

HMA Type	Wheel-Tracking Slope (mm/1000 Cycles)	Rut Depth (mm)
Reference	0.06	3.1
FC	0.18	5.7
AS	0.07	3.7

**Table 13:** Marshall and ITS tests summarized result.

Mixture Type	Marshall Stability (kN)	ITS (kN)		
		Unconditioned	Conditioned	TSR (%)
Reference	12.82 ± 0.39	158.82 ± 8.03	90.11 ± 7.31	56.73
Mixture AS	14.71 ± 0.20	173.58 ± 0.69	120.82 ± 0.76	69.61
Mixture FC	15.73 ± 0.73	170.77 ± 6.70	109.79 ± 3.54	64.29

## Data Availability

The datasets used and/or analyzed during the current study are available from the corresponding author on reasonable request.
